# Fibroblast growth factor 21 may be a strong biomarker for renal outcomes: a meta-analysis

**DOI:** 10.1080/0886022X.2023.2179336

**Published:** 2023-04-03

**Authors:** Guo Yong, Ling Li, Shanbiao Hu

**Affiliations:** a Department of Kidney Transplantation, The Second Xiangya Hospital of Central South University, Changsha, China; b Clinical Research Center for Organ Transplantation in Hunan Province, Changsha, China; c Department of Organ Procurement Organization, The Second Xiangya Hospital of Central South University, Changsha, China; d Department of Urinary Surgery, The Second Xiangya Hospital of Central South University, Changsha, China

**Keywords:** Fibroblast growth factor 21, chronic kidney disease, renal outcomes, meta-analysis

## Abstract

**Background:**

Fibroblast growth factor 21 (FGF21) is deemed to play an important role in kidney outcomes, while the association between FGF21 and various kidney diseases remains largely unclear and inconsistent. Therefore, we conducted this meta-analysis to find out the role of FGF21 in various renal diseases.

**Methods:**

The outcome indicator of our study was assessed by the pooled standard mean difference (SMD) with 95% confidence intervals (CIs) which were calculated by random-effect model analysis. The risk of bias was assessed by Non-Randomized Studies of Interventions (ROBINS-I) tool. Funnel plot combined with Egger’s and Begg’s tests was performed to estimate the publication bias that existed in the study.

**Results:**

A total of 28 eligible studies with 19348 participants were included in our research. The agreement between authors reached a kappa-value of 0.88. Overall, the serum FGF21 level was strongly higher in CKD patients (SMD = 0.97 (ng/L); 95% CI, 0.70—1.24 (ng/L)) and the renal outcomes in T2DM patients (SMD = 0.54 (ng/L); 95% CI, 0.39–0.70 (ng/L)) compared with the control group. Consistent with this, the incidence of CKD (OR = 2.56; 95% CI, 1.72–3.81) and the incidence of renal outcomes (OR = 1.63; 95% CI, 1.31–2.01) in T2DM patients was significantly higher in the patients with high FGF21 concentration, indicating that high serum FGF21 level may predict the incidence of CKD and the renal outcomes in T2DM patients.

**Conclusion:**

Serum FGF21 may be one of the strong predictors for various kidney diseases including the progression of CKD and the hard renal outcomes in type 2 diabetes patients, but more large-scale clinical research are needed to confirm this finding.

## Introduction

Fibroblast growth factor 21 (FGF21), a member of the fibroblast growth factor gene family, is a novel hepatoadipokine that is mainly produced from the liver and has been revealed as an important biomarker or metabolic regulator for various clinical diseases [1]. Actually, the administration of recombinant FGF21 would improve the dyslipidemia and weight loss in both animals and clinical diabetes patients [1,2]. Paradoxically, higher FGF21 level was observed in obesity-related diseases including nonalcoholic fatty liver disease [3], coronary heart disease [4], type 2 diabetes mellitus (T2DM) [5] and metabolic syndrome [6], which may be resulted from FGF21 resistance. A Chinese cohort also demonstrated that high circulating FGF21 would be a predictive factor for the incidence of T2DM [7].

In the context of renal diseases, high FGF21 has recently been revealed as one of the significant markers in various renal dysfunction including the progression of chronic kidney disease (CKD) [8,9] and the impaired glomerular filtration rate (GFR) and albuminuria in T2DM [10]. CKD, characterized by the destruction of the kidney structure and function resulted from various reasons [11], is a progressive disease with a high prevalence which reached to 10% in adults [12]. A large scaled Chinese population-based study showed a link between fatty liver disease and CKD [13]. Apart from this, more devastating consequences of CKD could be ignored, such as the synergistic effect on the progression of cardiovascular disease, anemia and bone disease, and other complications which would increase premature death [14]. Therefore, CKD presents a significant social burden, especially when comes to end-stage renal disease (ESRD), which must take regular dialysis or kidney transplantation to prolong survival time [15]. Considering the early symptoms of CKD remain noteless and the progression usually takes many years to be observed, early detection of CKD is urgent and important to help patients get early lifestyle and pharmacological treatments.

So far, GFR and albuminuria are the most commonly used method to estimate renal function, while the disadvantages of being time-consuming and difficulty in measuring GFR make them limited [16]. Several new biomarkers have been discovered to predict renal function impairment. Of note, FGF21 is one of the promising predictors of renal function. FGF21, a member of the fibroblast growth factor gene family, contributes to cell growth and differentiation, wound repair, and embryogenesis [17,18]. Although FGF21 was proven to play a beneficial effect on the healthy *via* its lipid-lowing, anti-oxidant, and anti-inflammatory properties, the circulating concentration of FGF21 is increased in various metabolic diseases [1]. Currently, FGF21 has been deemed as one of the emerging biomarkers of CKD disease [19]. Serum FGF21 concentration was found to be 20 times in CKD patients compared with a normal group [8]. Consistently, higher FGF21 level is also significantly associated with the incidence of proteinuria and ESRD in patients with type 2 diabetes [10,20]. Taken together, the existing evidence indicated that FGF21 may be a strong predictor for renal function.

To further confirm the relationship between FGF21 level and renal function, we conducted this meta-analysis on the value of FGF21 in predicting renal dysfunction in various diseases.

## Method

This meta-analysis was conducted rigorously performed according to the Preferred Reporting Items for Systematic Reviews and Meta-Analyses (PRISMA) statement guidelines, as previously described [21].

### Article Search strategy

We searched for included researches from 1 November 2021 to 20 December 2022. Four databases including PubMed (20 December 2013–2022), EMBASE (20 December 1960–2022), Cochrane Library (20 December 1960–2022) and Web of Science (20 December 1950–2022) were searched for eligible articles. All published articles related to both FGF21 and CKD or renal function were screened. The following terms were employed: ‘fibroblast growth factor 21’, ‘FGF21’, ‘renal’, ‘kidney’. The search strategy was demonstrated in Supplementary Table 1. Additional papers were identified by performing manual searches of the references of relevant articles and tracking citations to obtain more relevant studies. All articles published by 20 December 2022 with no language restrictions were included.

**Table 1.** Description of eligible studies reporting the association between FGF21 and CKD.

No	Author, year	Region	Race	Study type	Age	BMI	Size	The definition of CKD	Measurement type
1	Stein, 2008 [29]	Germany	Caucasian	Cross-section	63 ± 19	28.2 ± 5.6	120	CKD was defined as estimated glomerular filtration rate (eGFR) <60 mL/min/1.73 m2 using the Chronic Kidney Disease Epidemiology Collaboration equation.	ELISA
2	Han, 2010 [26]	South Korea	Asian	Cross-section	49.7 ± 7.0	33	135	end-stage renal disease patients receiving long-term peritoneal dialysis	ELISA
3	Lin, 2011 [9]	China	Asian	cohort	49.5 ± 12.3	21 ± 2.5	240	All patients were classified into chronic kidney disease (CKD) stages 1–5 according to the National Kidney Foundation–Kidney Disease Outcomes Quality Initiative (KDOQI) guidelines	ELISA
4	Crasto, 2012 [24]	USA	Caucasian	Cross-section	57.5 (50.2, 66.7)	25.7 (23.2, 29.4)	744	End-stage CKD	ELISA
5	Hindricks, 2014 [8]	Germany	Caucasian	cohort	55.3 ± 16.3	26.6 ± 6.1)	499	CKD patients were divided into three groups according to the eGFR, namely early-stage group (preserved renal function, eGFR 60 to 90 ml/min per 1.73 m2), middle-stage group (eGFR 30 to 60 ml/min per 1.73 m2) and end-stage group (hemodialytic group, eGFR,30 ml/min per 1.73 m2)	ELISA
6	Reinhard, 2015 [28]	Denmark	Caucasian	cohort	61 (39–74)	25.4 ± 1.2	24	hemodialysis (HD) patients.	ELISA
7	Kohara, 2017 [27]	Japan	Asian	cohort	66.1 ± 12.9	21.7 ± 3.0	90	Chronic hemodialy patients.	ELISA
8	Wu, 2018 [32]	China	Asian	cohort	65.3 ± 14.0	25.2 ± 4.1	531	Pediatric patients with CKD	ELISA
9	Sahapab, 2019 [35]	USA	Caucasian	cohort	60.3 ± 10.2	27.2 ± 5.0	5724	CKD without mentioned definition	ELISA
10	Myśliwiec, 2019 [36]	Poland	Caucasian	Cross-section	53.8 ± 13.3	26.6 ± 4.1	178	CKD patients with eGFR no more than 60 ml/min per 1.73 m2	ELISA
11	Zuzanna, 2020 [30]	Poland	Caucasian	Cross-section	10.7 ± 4.6	18.5 ± 4.7	63	CKD without mentioned definition	ELISA
12	Ángel, 2021 [31]	Mexico	Caucasian	Cross-section	52 ± 9	28.5 ± 5.4	382	Patients with stage 1-4 CKD	ELISA
13	Wei, 2021 [33]	China	Asian	Cross-section	6.84 ± 3.62	NA	31	A serum creatinine level >880 μmol/l or eGFR <15 ml/min/1.73m2 or dialysis treatment for >3 months, or prior kidney transplantation.	ELISA
14	Jiang 2021	China	Asian	Cross-sectional	57 ± 16	NA	802	Reaching eGFR <60mL/min/ 1.73 m2 with eGFR loss rate ≥ 1mL/min/1.73 m2 per year	ELISA

### Selection criteria

Two authors (YG and LL) independently reviewed all searched studies, and the determination of eligible researches were finalized. Disagreements were figured out through consensus or the help of a third reviewer (SH). All the articles included in this study met the following criteria: (1) they contain the information on FGF21 in the subjects with CKD; and (2) Studies including at least two groups, the CKD and control group. Articles were excluded if they met the following criteria: (1) articles lacking information or data necessary for the purpose of this article and (2) they were published as letters, editorials, reviews or conference abstracts.

### Data extraction

All relevant articles were imported into EndNote X9 software and reviewed independently by two authors (YG and LL). Discrepancies between authors were settled with the help of a third reviewer (SH). The Cohen’s kappa value was used to access the agreement during the systematic searches. The following information was extracted from the selected studies by two independent investigators: author, year, country, type of study, age, sample size, population and FGF21 levels. All the extracted data were then imported into Excel.

### Definition of the renal outcomes

Definition of the renal outcomes was carried out as previously described [22,23]. The renal outcomes were a composite of the decline in eGFR and or the worsening stage of albuminuria or the incidence of kidney disease. The decline in eGFR was defined as a loss of > 30% of kidney function compared with the value at baseline, and the decline should be confirmed by the consecutive test after 6 months. Albuminuria status was determined by the urinary albumin–creatinine ratio (UACR) and the patients were categorized according to the following stages: normoalbuminuria (UACR < 30 mg/g creatinine), microalbuminuria (UACR 30–300 mg/g creatinine), and macroalbuminuria (UACR > 300 mg/g creatinine). Progression of albuminuria was defined by progressive shifts in the albuminuria status, i.e. from normoalbuminuria to microalbuminuria, from microalbuminuria to macroalbuminuria, or from normoalbuminuria to macroalbuminuria. Progression of albuminuria was also confirmed by the consecutive result of UACR 6 months after the previous test.

### Statistical analysis

All analyses were performed using Stata (Version 13.0). The correlation between FGF21 levels and CKD or renal outcomes was expressed as the standardized mean difference (SMD) and 95% confidence interval (CI). A random-effects model was used for all results of our meta-analysis. I^2^ statistics were used to assess the degree of heterogeneity as follows: 25%, 50%, and 75% represented low, moderate, and high degrees of heterogeneity, respectively. Additionally, Begg’s and Egger’s tests and funnel plots were used to detect potential publication bias, with a *p*-value <0.05 suggesting the presence of bias.

## Results

### Search results and study characteristics

**Figure 1.**
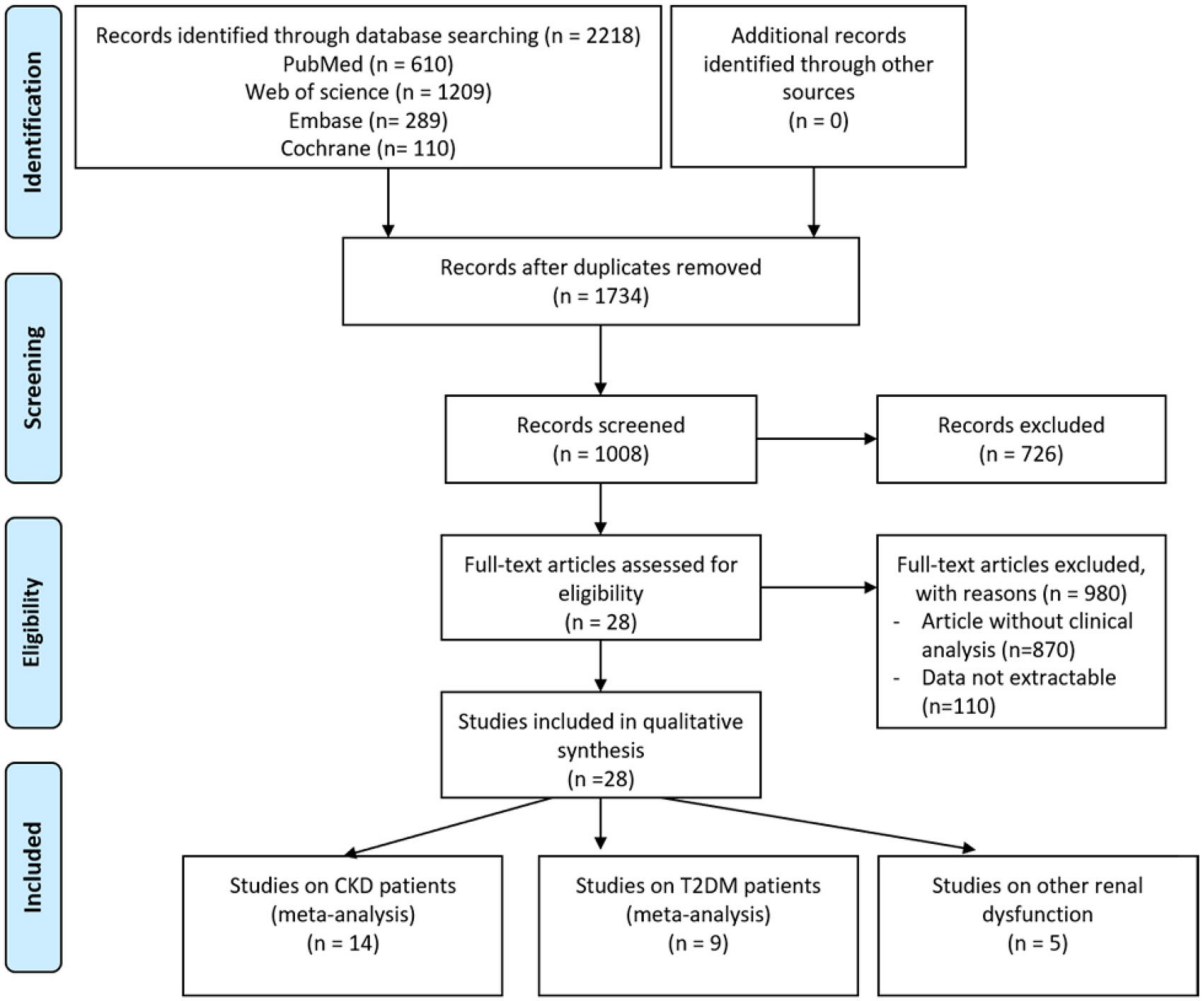
Flow diagram of the study selection process.

The flow diagram of the search progress was shown in Figure 1. A total of 2218 studies were identified after a preliminary search of the selected databases. After 484 duplicates were further removed, 1734 researches were screened by the titles and abstracts, of which 726 studies were further eliminated. Of the remained 1008 articles, 980 studies were unqualified for the following reasons after full-text reading: (1) articles without enough clinical data (*n* = 980); and (2) the data was unextractable (*n* = 110). Therefore, the final 28 articles [8,9,24–36] were included in our meta-analysis (Figure 1). The included study characteristics were divided by the kindness of renal diseases in Table 1–3. Among the 28 studies, 2 were performed in the USA, 3 were in China, 1 in Mexico, 1 in Singapore, 1 in South Korea, 2 in Germany, 1 in Japan, 1 in Demark, and 2 in Poland. All included articles consisted of observational studies including cohort and cross-sectional studies.

**Table 2.** Description of eligible studies reporting the association between FGF21 and CKD.

No	Author, year	Region	Race	Study type	Age	BMI	Size	Patients
1	Lee 2015	China	Asian	cross-section	57.2 ± 8.66	26.2 ± 4.64	1136	T2DM
2	Looker 2015	UK	Caucasian	Cohort	72 (66–76)	29.5 (26.1, 34.4)	207	T2DM
3	Xu 2016	China	Asian	cross-section	61.0 ± 4.0	27.4 ± 5.0	459	T2DM
4	Esteghamati 2017	Iran	Asian	cross-section	52.52 ± 8.99	26.42 ± 2.92	130	T2DM
5	Liu 2018	Singapore	Asian	Cohort	59.5 ± 10.2	26.4 ± 4.4	1700	T2DM
6	Zhang 2020	China	Asian	Cohort	56.0 ± 12.8	26.1 ± 3.9	2066	Hospitalized patients with type 2 diabetes
7	Zhang 2021	China	Asian	Cohort	56.6 ± 8.7	26.4 ± 3.7	2425	T2DM
8	Chang 2021	China	Asian	a prospective observational study	63.0 (54–70)	25.8 (23.5–28.4)	312	T2DM
9	Chang 2022	China	Asian	Cohort	61.2 ± 13.0	26.2 ± 4.5	312	T2DM

**Table 3.** Description of eligible studies reporting the association between FGF21 and other renal diseases.

No	Author, year	Region	Study type	Age	BMI	Size	Patients	Findings
1	Bagheri 2016	Iran	cross-section	40.16 ± 10.19	25.30 ± 3.84	86	Kidney transplant Patients	FGF21 was positively related with the dialysis time of kidney transplant patients
2	Trakarnvanich 2017	Thailand	cross-section	47.24 ± 11.80	24.67 ± 4.19	90	Kidney transplant Patients	FGF21 was negtively related with the renal function in kidney transplant patients
3	Wu 2018	China	cohort	65.3 ± 14.0	25.2 ± 4.1	531	Patients undergoing coronary Angiography	Higher FGF21 concentration had higher incidence of CKD and renal function decline in patients undergoing coronary angiography
4	Post 2021	Netherlands	cohort	65 ± 15	25.5 ± 4.3	59	hemodialysis patients	Higher plasma FGF21 is associated with higher odds of low protein intake in hemodialysis patients.
5	Matsui 2021	Japan	cross-sectional	64 ± 9	22.5 ± 3.5	272	hemodialysis patients	Elevated circulating FGF21 levels partially mediate the association of elevated blood pressure and/or aortic stiffness with renal dysfunction in middle-aged and older adults

### Quality assessment

Quality assessment was performed among each included study by ROBINS-I. The results of the included studies in this meta-analysis were at moderate risk.

### FGF21 level in CKD patients

**Figure 2.**
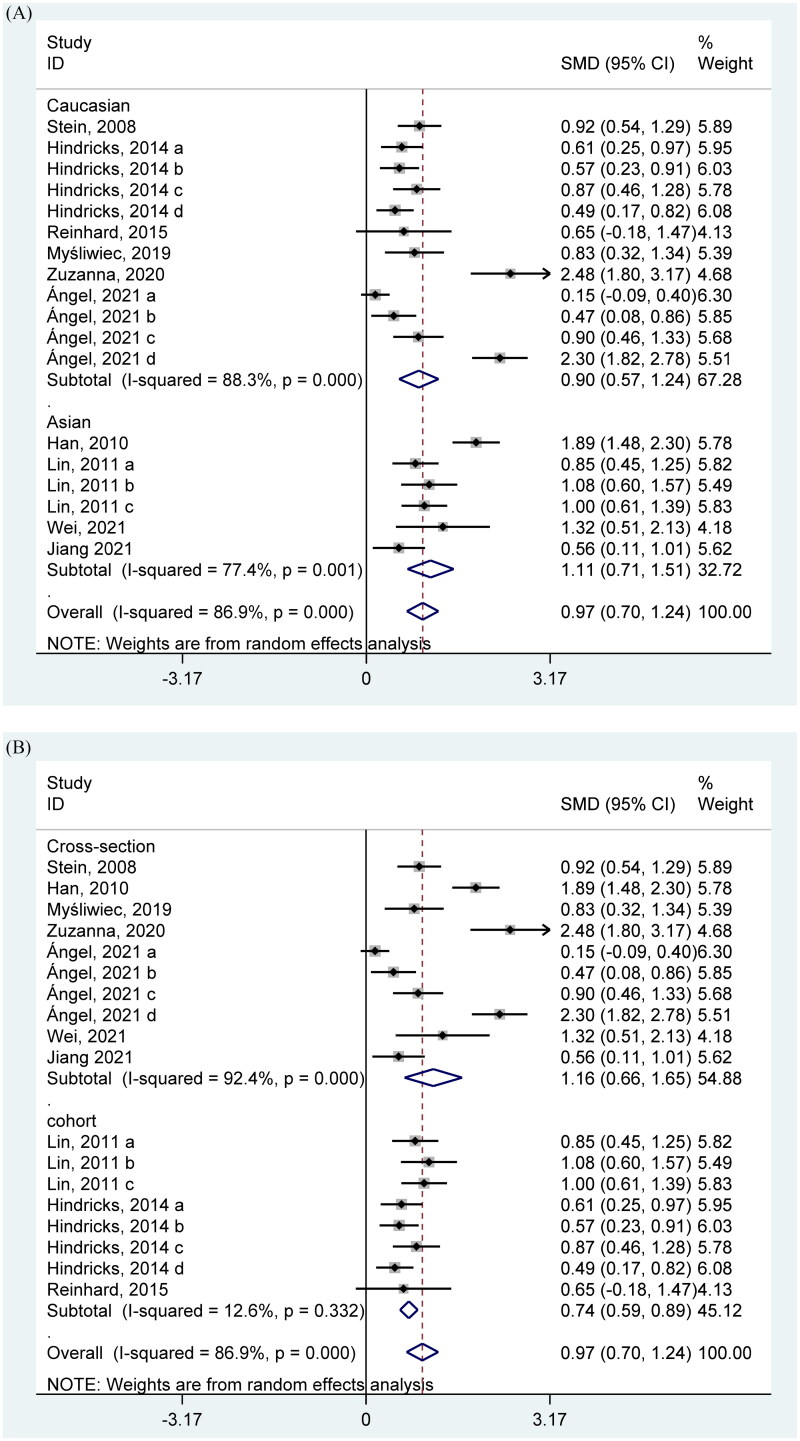
Forest plots of SMD for the association between FGF21 and CKD.

A total of 14 studies were included in the comparison of FGF21 levels in CKD and control groups. In general, FGF21 level was significantly increased in the CKD patients compared with the control (SMD = 0.97 (ng/L) 95% CI, 0.70–1.24 (ng/L)) with high heterogeneity (I^2^ = 85.5%, *p* < 0.001) (Figure 2). Strong heterogeneity remained high in the subgroup analysis which was based on race (Figure 2(A)), while the high heterogeneity vanished in the cohort subgroup although remain similar in the cross-sectional study (Figure 2(B)). Both the results of funnel analysis (Supplementary Figure 1(A)) and Egger’s test (*p* = 0.009) showed the abnormal distribution, the Begg’s test also (*p* = 0.009) demonstrated that the publication bias may exist in this part. However, the trim and fill analysis were further conducted, and found that our results were stable (Supplementary Figure 1(B)). Additionally, the sensitivity analysis performed by excluding one study one time did not change our results (Supplementary Figure 1(C)), indicating that our results remain stable.

### High FGF21 and CKD incidence

**Figure 3.**
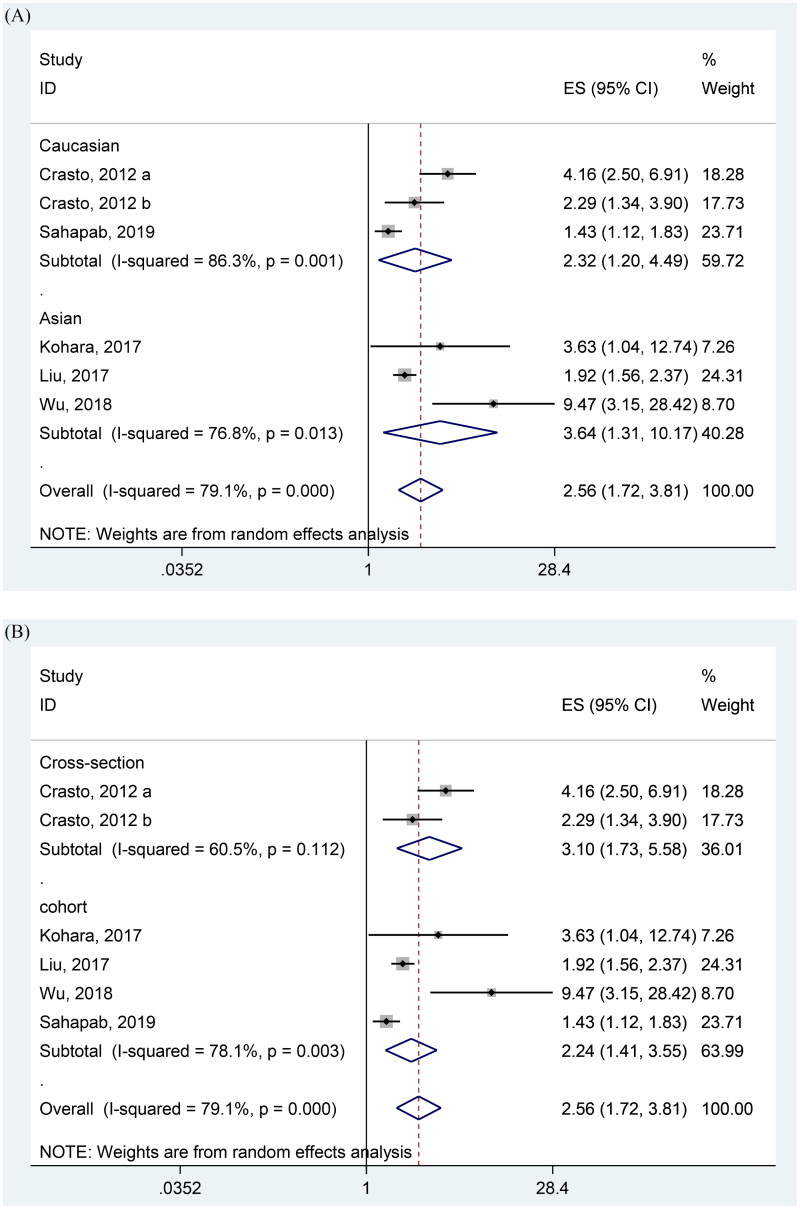
Forest plots of odds ratios (OR) for the association between the FGF21 and CKD.

Overall, 5 studies have reported related data concerning the effect of high FGF21 on CKD morbidity. Consistent with the result of the high FGF21 level in CKD patients (Figure 2), the risk of CKD was higher in the high FGF21 group compared with the normal group (OR = 2.56 95% CI, 1.72–3.81) (Figure 3). Regarding the results of the funnel analysis (Supplementary Figure 2(A)), Egger’s test (*p* = 0.079), and Begg’s test (*p* = 0.452), the publication bias did not exist. The sensitivity analysis also revealed that this result was stable (Supplementary Figure 2(B)).

### FGF21 level in T2DM patients with positive renal outcomes

**Figure 4.**
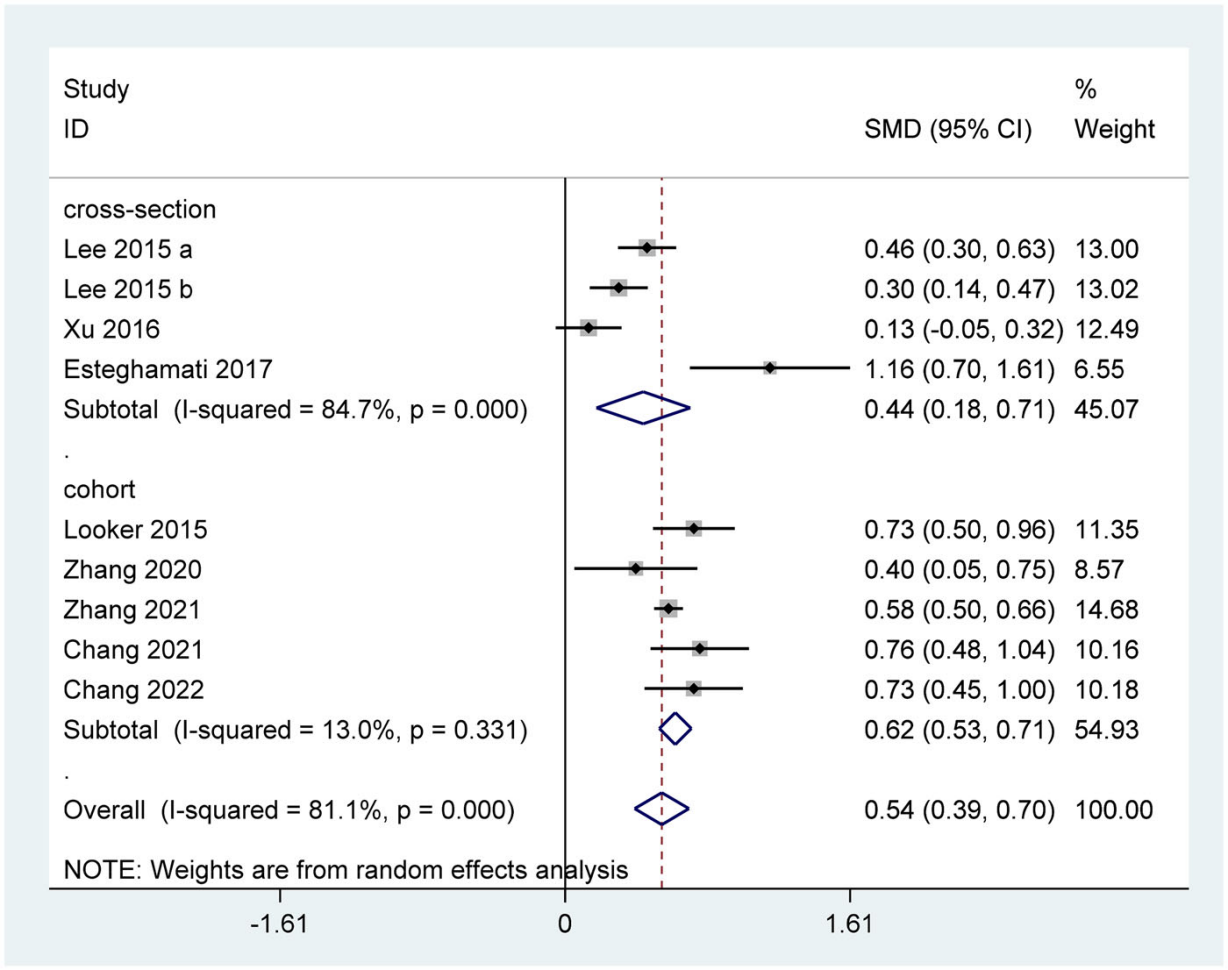
Forest plots of SMD for the association between FGF21 and the renal dysfunction in T2DM patients.

Overall, FGF21 level was significantly increased in T2DM with positive renal outcomes (SMD = 0.54 (ng/L) 95% CI, 0.39–0.70 (ng/L)) (Figure 4). Although Begg’s test (*p* = 1.00) showed no statistical significance, both the results of the funnel plot (Supplementary Figure 3(A)) and the Egger’s test (*p* = 0.048) revealed that there is publication bias exists in this result. The trim and fill analysis were further conducted and found our results may be still stable (Supplementary Figure 3(B)). The sensitivity analysis by removing one study at a time showed that two studies are inconsistent with the results, while as the limited included studies, we have not further excluded these two results (Supplementary Figure 3(C)).

### High FGF21 and the positive renal outcomes of T2DM patients

**Figure 5.**
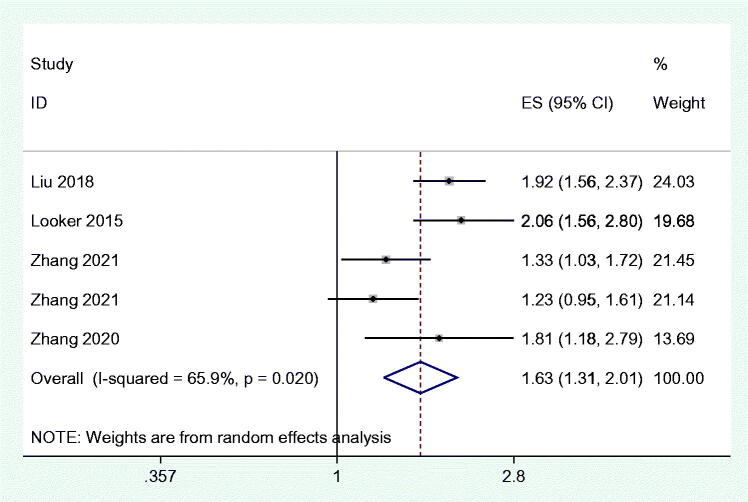
Forest plots of odds ratios (OR) for the association between the FGF21 and the renal dysfunction in T2DM patients.

Only 4 eligible studies with 5 results have mentioned the effect of high FGF21 levels on the incidence of renal outcomes in T2DM patients. All the included studies in this part were cohort designs. In detail, circulating FGF21 was positively associated with the increasing prevalence of renal outcomes in T2DM (Figure 5). Egger’s test (*p* = 0.979) and Begg’s test (*p* = 0.806) showed no statistical significance. Consistently, the funnel plot also showed symmetric distribution (Supplementary Figure 4(A)). Sensitivity analysis also proves the stability of our results (Supplementary Figure 4(B)).

### Association between FGF21 and renal outcomes in other diseases

There are three researches contained the association between FGF21 and renal outcomes in other diseases (Table 3). For details, a China cohort revealed that higher FGF21 levels increased renal function decline in patients undergoing coronary angiography [32]. Additionally, two studies have found that FGF21 was inversely related to renal function in kidney transplant patients [37,38]. As the limited eligible studies, no further meta-analysis was performed for this part.

## Discussion

### Association between FGF21 and kidney diseases

Our meta-analysis conducted to determine the association between FGF21 and renal function included various renal diseases such as CKD and diabetic nephropathy, which provides sufficient evidence for the inverse relationship between high FGF21 and the health of the kidney. In detail, the FGF21 level was significantly higher in CKD patients (Figure 2). High FGF21 concentration was also positively related with the wore outcomes of the renal function and incidence of CKD (Figure 3). In the context of the renal function of T2DM patients, higher FGF21 concentration was observed in the T2DM with positive renal outcomes than the T2DM with normal renal function (Figure 4). Consistently, circulating FGF21 levels also increased the incidence of renal outcomes in T2DM patients (Figure 5).

### Mechanism underlying the relationship between FGF21 and renal function

The mechanisms accounting for the association between FGF21 and renal function remain unclear, while the following mechanisms may partly explain the phenomenon. Firstly, emerging solid evidence has demonstrated the beneficial role of FGF21 in preventing diet-induced obesity [39], weight loss, and improved glucose tolerance [40]. Moreover, administration of FGF21 would result in reversed diabetic characteristics including reduced fasting glucose, as well as declined LDL and TG cholesterol levels [41]. In detail, FGF21 treatments in obese mice showed significantly higher energy expenditure, lipid excretion, and fat utilization [42]. Therefore, we speculated that increased circulating FGF21 levels may be one marker of the compensatory ability of the renal outcomes to protect from adverse effects such as vascular and metabolic diseases.

Another hypothesis of the potential role of FGF21 in renal outcomes may be FGF21 resistance, which is similar to insulin resistance in obesity and type 2 diabetes. FGF21 resistance has previously been described in obesity, of which the mRNA level of FGF21 was observed higher in the white adipose and liver tissues of the obese mice [43]. However, more animal studies are needed to examine the exact mechanism underlying the FGF21 resistance in renal outcomes.

Thirdly, one clinical study [6] has revealed that circulating FGF21 level was positively associated with increased waist-to-hip ratio (WHR) and waist-to-height ratio (WHtR). which were the markers of the adverse body fat distribution. Consistent with this, another article also indicated the positive relationship between FGF21 and WHR [8]. Considering the indicative function of the WHR and WHtR in the adverse body fat distribution, we speculated the high circulating FGF21 state may be contributed by the accumulation of visceral fat or the adverse fat composition.

### Theoretical and practical significance

The theoretical aim of our study was mainly to determine FGF21 as the prognosis factor in the clinical diagnosis. Consistent with the hypothesis, FGF21 was greatly higher in the patients suffering from renal diseases and high FGF21 increased the incidence of CKD disease and the renal outcomes in type 2 diabetes patients. Therefore, FGF21 could be a strong biomarker of early diagnosis and treatment for CKD and positive renal outcomes. For the research significance, our study has pointed out the necessity and urgency for researching the potential pathogenic effects of FGF21 in renal diseases.

### Limitations of the study

There are following limitations existing in our meta-analysis. First, although the results of the relationship between FGF21 level in renal function were consistent, the high heterogeneity in our results still remained, while we performed the subgroup analysis and found that cohort analysis significantly decreased the heterogeneity in the subgroup analysis. Additionally, although the publication bias remained in the primary analysis of some parts of the results, the trim and fill analysis was performed, and found that our results were stable. And the study design of the included studies contained cross-sectional and cohort design, it is known that a cross-sectional study cannot establish a causal relationship between FGF21 and kidney disease. Although the outcome of the subgroup based on study design demonstrated that both cross-section and cohort study showed the inverse relationship between FGF21 and risk of renal disease, a more well-designed cohort is urgent to improve or deny our findings. Second, the number of eligible studies on the association between high FGF21 levels and other renal disease outcomes was very limited, leading to the instability of the conclusion of this part. Additionally, our study has not registered in the PROSPERO in advance, which is also one of our limitations.

## Conclusion

In summary, our study discovered that FGF21 was significantly associated with worse renal outcomes in various renal diseases including CKD and diabetic nephropathy. Further clinical studies were needed to determine the critical role of FGF21 in renal outcomes to provide a more therapeutic target for renal diseases.

## Supplementary Material

Supplemental Material

Supplemental Material

## Data Availability

All data generated or analyzed during the present study are included in this published article.
