# Supplementary material for: Fibroblast growth factor 21 may be a strong biomarker for renal outcomes: a meta-analysis
Source: Ren Fail. 2023 Apr 3;45(1):2179336. doi: 10.1080/0886022X.2023.2179336 (PMC10071947; doi:10.1080/0886022X.2023.2179336)
Supplement: Supplemental Material [file IRNF_A_2179336_SM6509.pdf]

No	Study	Bias due to confounding	Bias in seletion of participants into the study	Bias in classification of interventions	Bias due to deviations from intended interventions	Bias due to missing data	Bias in measurement of outcomes	Bias in selection of the reported results	Overall bias
1	Candace Crasto, 2012	low	low	moderate	moderate	low	low	moderate	moderate
2	Han, 2010	moderate	moderate	low	low	moderate	moderate	moderate	moderate
3	Janka Hindricks, 2014	moderate	moderate	moderate	moderate	moderate	low	moderate	moderate
4	Marina Kohara, 2017	low	moderate	low	moderate	low		low	moderate
5	Zhuofeng Lin, 2011	moderate	low	moderate	low	moderate	low	moderate	moderate
6	Mark Reinhard, 2015	low	low	low	moderate	moderate	moderate	moderate	moderate
7	Sebastian Stein, 2008	low	moderate	low	low	low	moderate	moderate	moderate
8	Zuzanna, 2020	moderate	low	moderate	low	low	low	low	moderate
9	Ángel, 2021	low	low	low	moderate	low	moderate	low	moderate
10	Myśliwiec, 2019	low	moderate	low	low	moderate	low	low	moderate
11	Wei, 2021	moderate	low	moderate	low	low	low	moderate	moderate
12	Wu, 2018	low	moderate	low	moderate	moderate	low	moderate	moderate
13	Liu, 2017	low	moderate	low	moderate	low	moderate	low	moderate
14	Sahapab, 2019	moderate	low	moderate	low	moderate	low	low	moderate
15	Lee 2015	low	moderate	low	low	low	moderate	moderate	moderate
16	Looker 2015	low	moderate	low	moderate	moderate	low	moderate	moderate
17	Xu 2016	moderate	moderate	low	low	moderate	moderate	moderate	moderate
18	Esteghamati 2017	moderate	low	moderate	low	moderate	low	moderate	moderate
19	Liu 2018	low	moderate	low	moderate	moderate	low	moderate	moderate
20	Zhang 2020	low	low	moderate	moderate	low	low	moderate	moderate
21	Zhang 2021	low	low	moderate	moderate	low	low	moderate	moderate
22	Chang 2021	moderate	low	moderate	low	moderate	low	moderate	moderate
23	Chang 2022	low	moderate	low	low	low	moderate	moderate	moderate

Information Classification: General

24	Bagheri 2016	moderate	low	moderate	low	moderate	low	moderate	moderate
25	Trakarnvanich 2017	moderate	low	moderate	low	moderate	low	moderate	moderate
26	Wu 2018	low	moderate	low	moderate	moderate	low	moderate	moderate
27	Adrian Post 2021	low	low	low	moderate	low	moderate	low	moderate
28	Masahiro Matsui 2021	moderate	moderate	moderate	moderate	moderate	low	moderate	moderate

Supplementary table 1: Risk of Bias in Nonrandomized studies of Interventions (ROBINS-I) tools of the included studies

Information Classification: General
