# Supplementary figures and images for: Fibroblast growth factor 21 may be a strong biomarker for renal outcomes: a meta-analysis
Source: Ren Fail. 2023 Apr 3;45(1):2179336. doi: 10.1080/0886022X.2023.2179336 (PMC10071947; doi:10.1080/0886022X.2023.2179336)

Figure

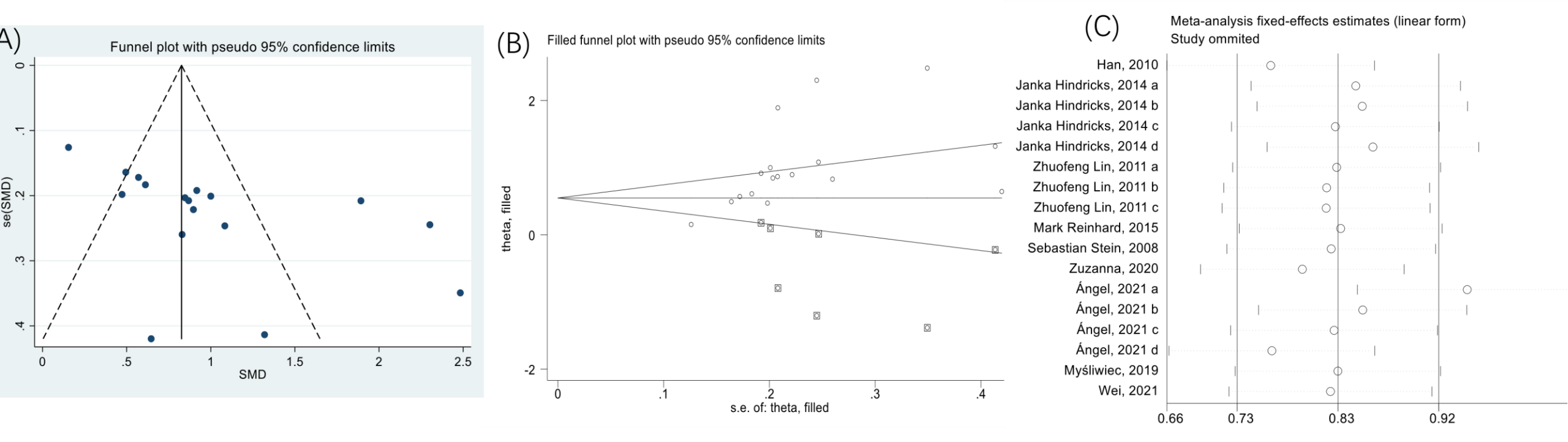

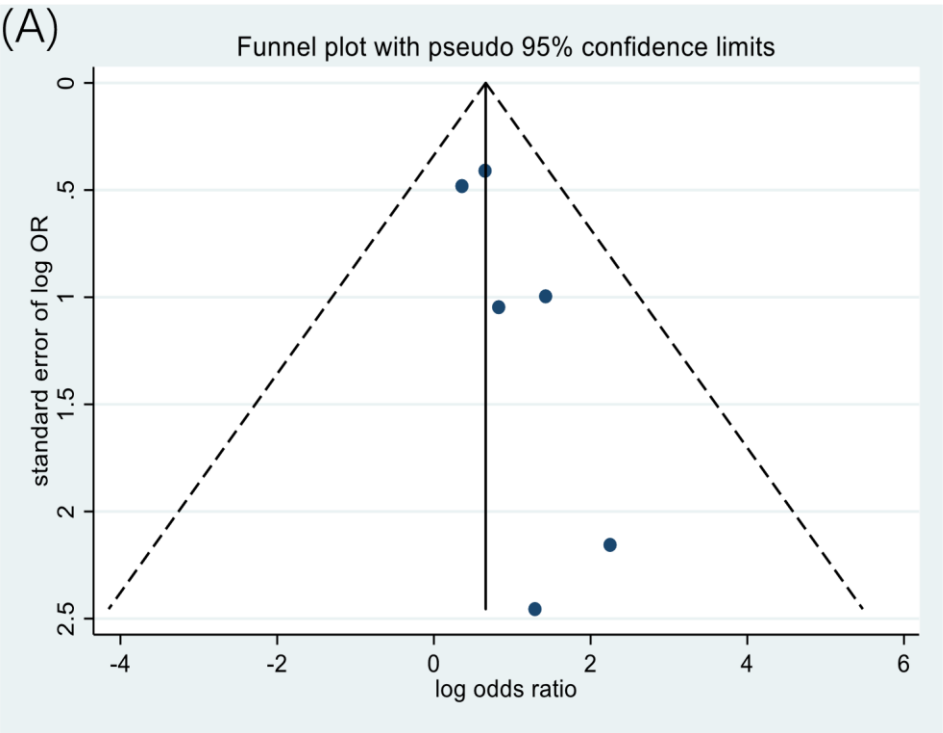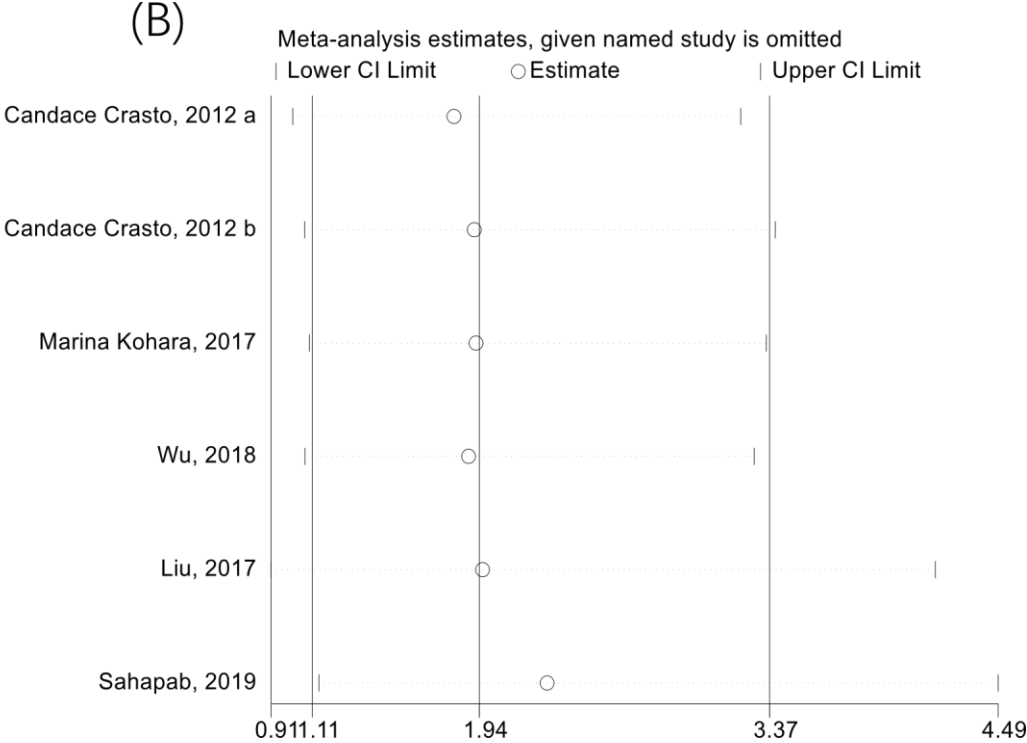

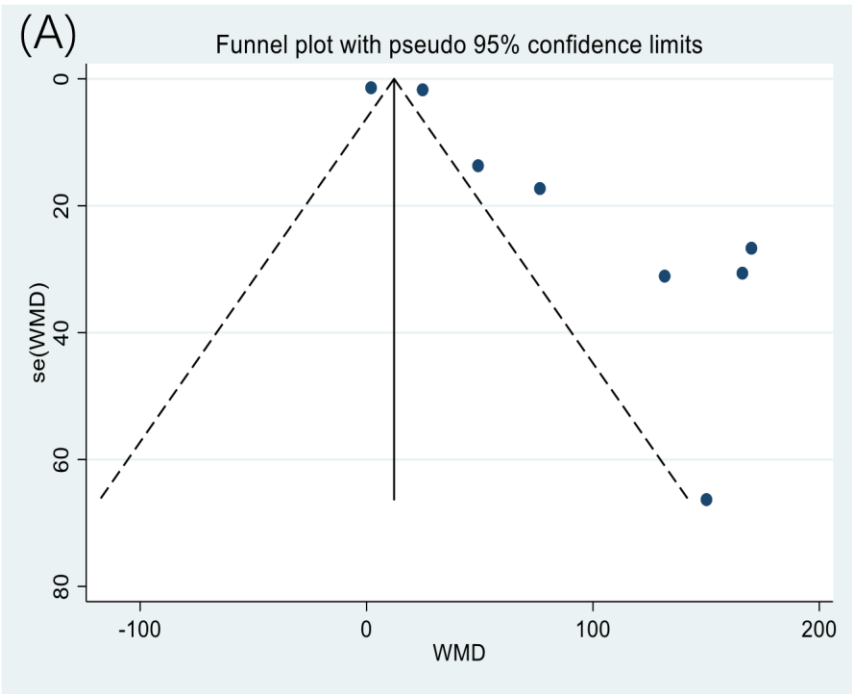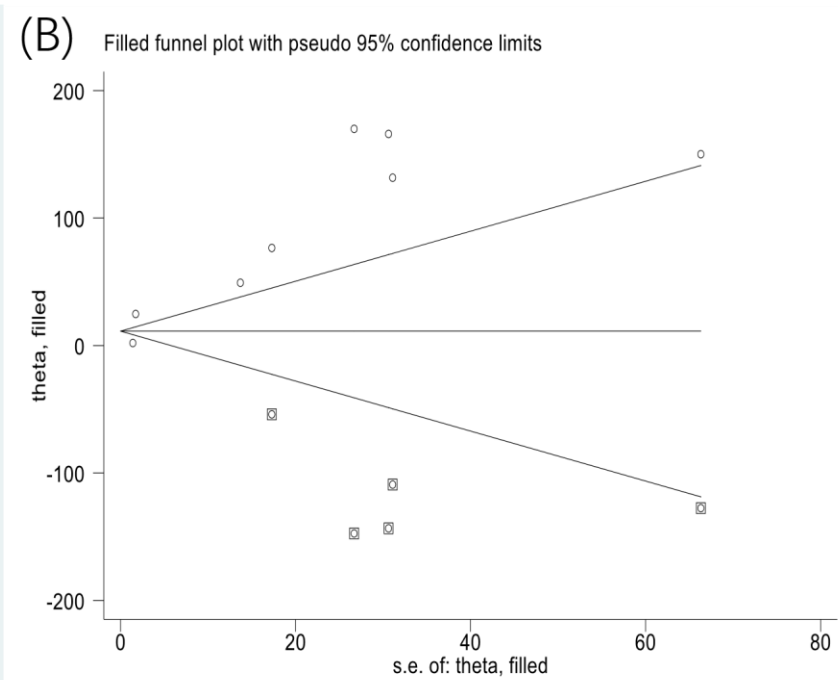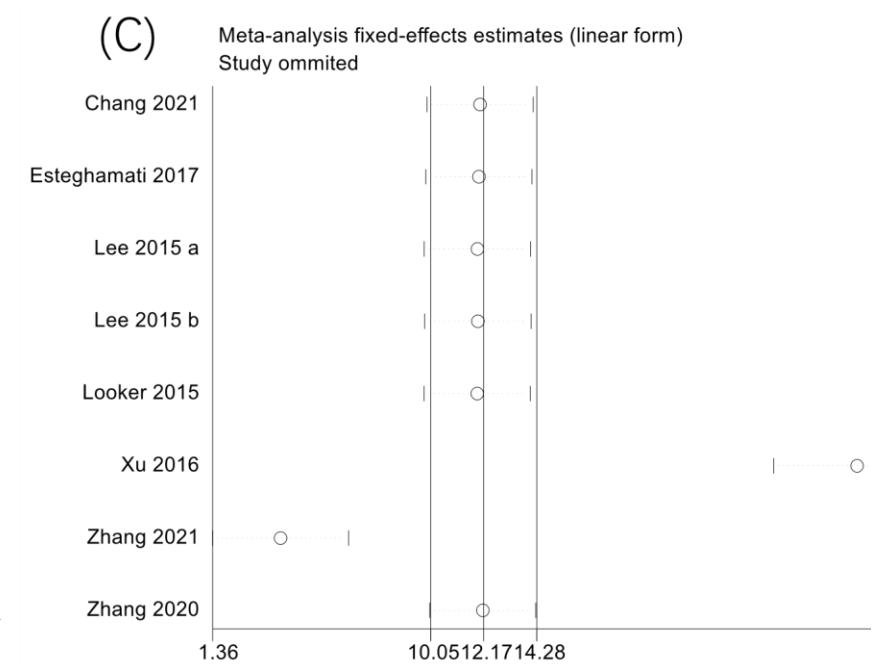

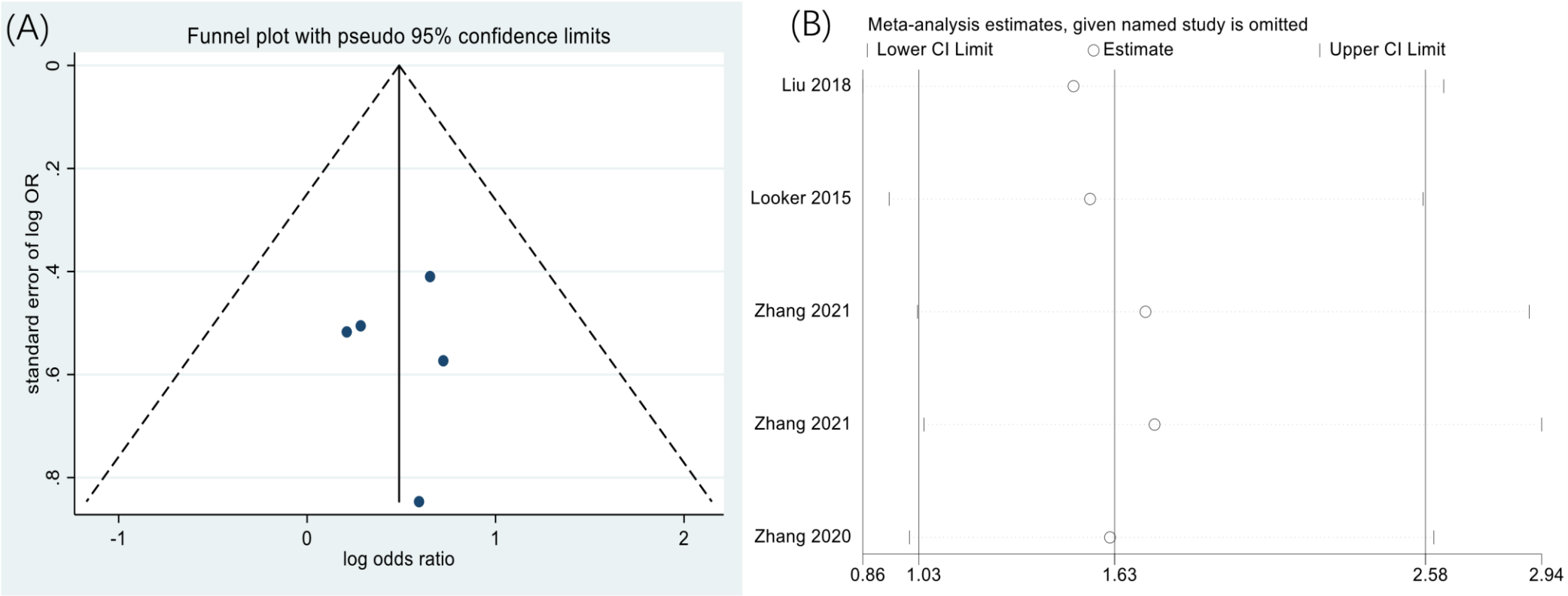

Supplement: Supplemental Material [file IRNF_A_2179336_SM6486.pdf]
